# Sweet syndrome in a patient receiving encorafenib and binimetinib therapy for malignant melanoma

**DOI:** 10.1016/j.jdcr.2024.01.025

**Published:** 2024-02-04

**Authors:** Myiah Quach, John P. Antonelli, Charlotte LaSenna, Mackenzie Asel, Jennifer Pleva, Vincent T. Ma

**Affiliations:** a University of Wisconsin School of Medicine and Public Health, Madison, Wisconsin; b Department of Dermatology, University of Wisconsin–Madison, Madison, Wisconsin; c UW Health, Madison, Wisconsin; d Division of Hematology, Medical Oncology and Palliative Care, Department of Medicine, University of Wisconsin–Madison, Madison, Wisconsin

**Keywords:** binimetinib, encorafenib, immune-related adverse events, melanoma, Sweet syndrome

## Introduction

Approximately 30% to 50% of malignant melanoma tumors contain activating oncogenic mutations in the BRAF gene which mediates cell growth through the RAS-MEK-ERK pathway. BRAF inhibitors have demonstrated antitumor effect by preventing downstream signaling that leads to tumorigenesis, but melanoma cells will often compensate by activating alternative signaling pathways within the MAPK pathway such as MEK.^1^ This resistance mechanism limits the long-term clinical efficacy of BRAF targeted therapy, but the addition of MEK inhibitors has been shown to prolong treatment resistance.^2^ Other disadvantages associated with anti-BRAF monotherapy are cutaneous toxicities and risk of secondary nonmelanoma skin cancers.^3^^,^^4^

Sweet syndrome, also known as acute febrile neutrophilic dermatosis, is associated with a variety of underlying conditions, and may be induced by medications. Diagnostic criteria require abrupt onset of painful, erythematous nodules or plaques and histopathologic findings of dense neutrophilic infiltrate of the dermis. This may be accompanied by pyrexia and abnormalities in laboratory tests such as erythrocyte sedimentation rate >20 mm/h, high C-reactive protein, leukocyte count >8000, or >70% neutrophils.^5^ In this case report, we describe the clinical course and outcome of a patient with Sweet syndrome in the context of encorafenib and binimetinib in the treatment of metastatic melanoma.

## Case summary

A female patient in her 30s was diagnosed with metastatic (stage IV) BRAF V600E-mutated melanoma with nodal, liver, lung, and cutaneous involvement in February 2022. She received 1 cycle of ipilimumab/nivolumab therapy in February 2022 that was complicated by immune-mediated hypothyroidism, colitis, and hepatitis requiring glucocorticoid therapy. Restaging positron emission tomography/computed tomography in March 2022 revealed worsening metastases and she was switched to BRAF/MEK targeted therapy.

**Fig 1:**
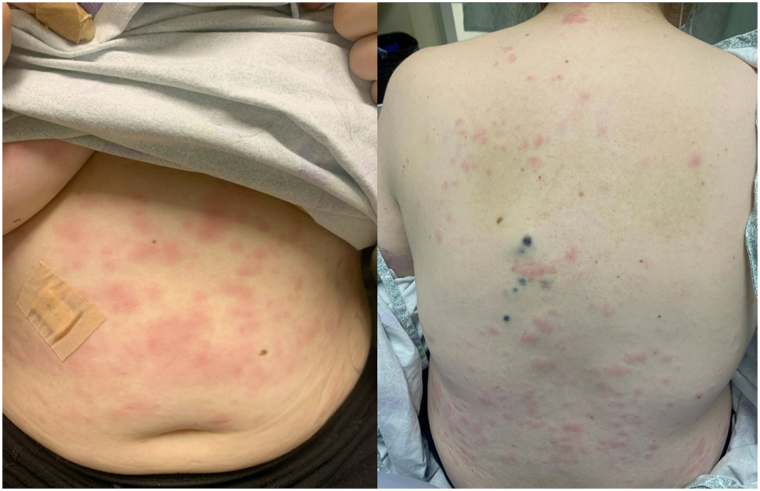
Examination revealed well-demarcated, erythematous, edematous papules and plaques on trunk and extremities. Cutaneous melanoma metastases were also noted on the left side of the mid portion of the back.

**Fig 2:**
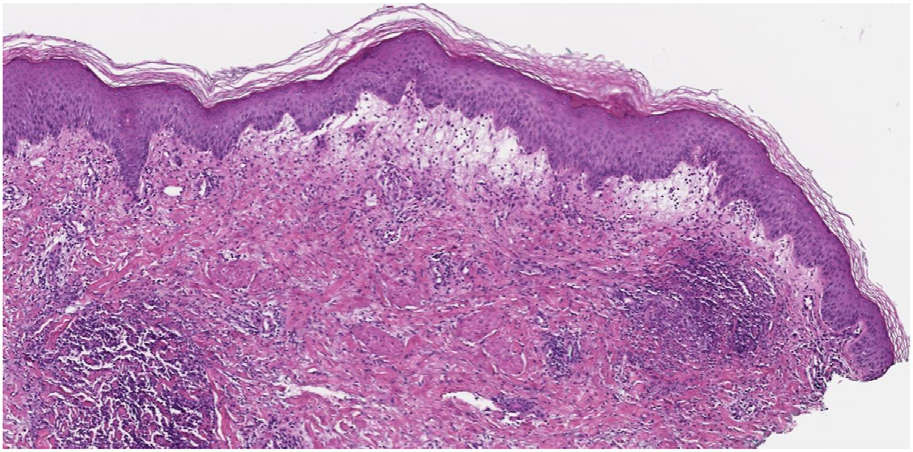
A punch biopsy of the patient’s pustular rash shows brisk papillary edema and collections of neutrophils in the dermis.

In April 2022, the patient started encorafenib (450 mg daily) and binimetinib (45 mg twice daily). Restaging positron emission tomography/computed tomography in June 2022 showed improvement in her known nodal, liver, and lung metastases. Her treatment course was complicated by recurrent immune-mediated colitis, *Streptococcus bovis* bacteremia with endocarditis, and immune-mediated hepatitis flare requiring hospitalization in August 2022. In September 2022, she presented to the emergency department because of fevers, chills, weakness, nausea, vomiting, and new onset rash. Her 4-week course of ceftriaxone for bacteremia was near completion, blood cultures were negative, and antibiotics were discontinued. On examination, she initially had pink thin plaques on the trunk, thighs and upper portion of the arms which became more edematous and tender over the course of 3 days (Fig 1), along with acneiform papules/pustules on the upper portion of the back and upper portion of the chest and superficially desquamating scale on some fingers. Bacterial culture and herpes simplex virus/varicella-zoster virus polymerase chain reaction of the pustular lesions were negative. A punch biopsy was performed of the pustular rash which showed brisk papillary dermal edema and collections of neutrophils in the dermis (Fig 2). Upon further history taking, the patient reported frequently forgetting her evening dose of binimetinib before admission. Treatment included withholding of encorafenib and binimetinib, continuous use of prednisone dosed at 1 mg/kg/d, and topical triamcinolone 0.1% twice daily. The rash improved quickly and resolved at the time of outpatient follow-up 1 week later. She was discharged on a prednisone taper of 10 mg/d every 7 days. The patient’s myriad of presenting symptoms and histopathologic findings supported a diagnosis of Sweet syndrome.

Encorafenib and binimetinib were restarted at 33% dose reduction in September 2022 and then increased to standard dose because of disease progression in October 2022. There was no recurrence of Sweet syndrome. A few months later, the patient developed progression of metastatic melanoma. She was transitioned to hospice and died soon after.

## Discussion

The pathomechanism of Sweet syndrome is not well understood, likely related to the diversity of underlying associations. It is thought to be triggered by an inciting event which leads to maturation and proliferation of neutrophils along with cytokine-mediated dermal localization.^5^ In our case, a drug-induced etiology was favored, possibly exacerbated by underdosing of the MEK inhibitor. A paraneoplastic phenomenon in the setting of metastatic melanoma could not be excluded but is considered less likely given rapid resolution of her rash with steroids despite progression of her malignancy. Her history of metastatic melanoma, antibiotic use, and prior immunotherapy likely contributed to the underlying immune dysregulation possibly exacerbated by a brief period of unopposed BRAF inhibition leading to the development of Sweet syndrome.

Other neutrophilic dermatoses were considered such as neutrophilic eccrine hidradenitis which clinically presents similarly to Sweet syndrome, but the distribution of this patient’s rash (including sparing of the face and palms), lack of preceding chemotherapy, and histopathology (distribution of the neutrophilic infiltrates throughout the dermis) were not fitting with neutrophilic eccrine hidradenitis.^6^

Paradoxical activation of MAPK by unopposed BRAF inhibition commonly leads to keratinocyte hyperproliferation seen in cutaneous squamous cell carcinoma.^7^ Combination of BRAF inhibitor with MEK inhibitor therapy decreases the squamoproliferative side effects seen in monotherapy but still produces cutaneous side effects.^7^ As of 2023, there are currently 3 Food and Drug Administration–approved combination therapies of BRAF/MEK inhibitors for the treatment of metastatic melanoma: (1) dabrafenib plus trametinib, (2) vemurafenib plus cobimetinib, and (3) encorafenib plus binimetinib. Sweet syndrome and histiocytoid Sweet syndrome have been reported in the context of dabrafenib plus trametinib in 2 cases reports,^8^^,^^9^ and neutrophilic dermatoses (including neutrophilic panniculitis) have been associated with vemurafenib monotherapy in at least 4 cases^10^ but not with encorafenib plus binimetinib.

Management of drug-induced Sweet syndrome involves cessation of the offending drug, if possible, with or without glucocorticoids.^5^ In this case, combined BRAF/MEK targeted therapy was resumed without reemergence of Sweet syndrome. This reinforces the hypothesis that BRAF inhibition is not eliciting a true hypersensitivity reaction but rather an erythematous eruption related to downstream activation of MAPK.^8^ Understanding the molecular basis of BRAF inhibitor-induced toxic effects is important for developing and choosing agents for cancer therapy and for elucidating the role of MAPK pathway in cutaneous homeostasis.

## Conflicts of interest

Dr Ma serves as a consulting or advisory board member for Immunocore, Regeneron Pharmaceuticals, Teiko.bio, Incyte, Replimune, Partner Therapeutics, and Bristol Myers Squibb. The other authors have no conflicts of interest to declare.
